# Impact of once-daily versus multiple-daily dosing of gentamicin on the incidence of acute kidney injury in patients treated with synergistic combinations of antibiotics

**DOI:** 10.1186/s40780-024-00360-y

**Published:** 2024-07-15

**Authors:** Kyohei Sugiyama, Keita Hirai, Yukako Suyama, Masato Tsutsumi

**Affiliations:** 1 Department of Pharmacy, Shizuoka General Hospital, 4-27-1 Kita Ando Aoi-ku, Shizuoka, 420- 8527 Japan; 2 Department of Clinical Pharmacology and Therapeutics, Shinshu University Graduate School of Medicine, 3-1-1 Asahi, Matsumoto, Nagano, 390-8621 Japan; 3 Department of Pharmacy, Shinshu University Hospital, 3-1-1 Asahi, Matsumoto, Nagano, 390- 8621 Japan

**Keywords:** Gentamicin, Infective endocarditis, Synergistic effects, Therapeutic drug monitoring, Acute kidney injury

## Abstract

**Background:**

Gentamicin is a commonly used antibiotic with synergistic effects that is administered once or multiple times daily. However, the influence of the daily administration frequency on renal function has not yet been identified. This study aimed to investigate the effect of the daily dosing frequency on worsening renal function in patients receiving gentamicin.

**Methods:**

This study included 35 patients undergoing gentamicin treatment who had at least one serum trough level measured and underwent therapeutic drug monitoring (TDM). We evaluated the influence of daily dosing frequency on gentamicin trough concentration and the risk of acute kidney injury (AKI).

**Results:**

Compared to patients who received gentamicin once-daily dosing (*n* = 22), patients who received multiple-daily dosing (*n* = 13) had significantly higher initial and minimum trough concentrations after TDM. The proportion of patients with trough concentrations lower than 1.0 µg/mL was significantly higher in the once-daily dosing group at the initial trough concentration, whereas there was no significant difference at the minimum trough concentration after TDM. AKI developed in nine patients; however, there was no significant difference in the incidence of AKI according to the frequency of daily gentamicin dosing. In contrast, a higher minimum trough concentration after TDM was found to be a risk factor for AKI development with an odds ratio of 9.2 (95% confidence intervals; 1.3–65.5).

**Conclusion:**

A higher trough concentration of gentamicin correlated with a higher incidence of AKI. The risk of developing AKI may be reduced by choosing a once-daily dosing regimen or implementing TDM.

**Supplementary Information:**

The online version contains supplementary material available at 10.1186/s40780-024-00360-y.

## Background

Practical and safe treatment of severe infections such as infective endocarditis (IE) is essential [1]. Gentamicin (GM) is effective against IE when used in combination with β-lactams or cell wall synthesis inhibitors such as vancomycin [2]. Because of the potential synergistic effect of this treatment, it is recommended by guidelines of the American Heart Association (AHA) [3], European Society of Cardiology (ESC) [4], British Society for Antimicrobial Chemotherapy (BSAC) [5], and Japanese Circulation Society (JCS) [6]. All guidelines recommend using GM for 2–6 weeks to achieve synergistic effects. However, the recommended dosages and administration methods differed among the guidelines. For example, the JCS guideline recommended a once-daily dosing of GM at 2 to 3 mg/kg/day. On the other hand, the AHA guidelines recommend once to three times daily dosing at 3 mg/kg/day, the ESC guidelines recommend once to twice-daily dosing at 3 mg/kg/day, and the BSAC guidelines recommend twice-daily dosing at 2 mg/kg/day. These difference in dosing frequency leads to complications in the therapeutic use of GM. GM is also administered for synergistic effects not only in the treatment of IE, but also in the treatment of patients infected with Gram-negative bacteria such as *Pseudomonas aeruginosa*, and guidelines recommend a once-daily dose of 3 mg/kg/day [7].

It has been recognized that elevated GM trough concentration is a risk factor for renal dysfunction [8, 9] and guidelines recommend maintaining a trough concentration of less than 1.0 µg/mL. Therefore, therapeutic drug monitoring (TDM) was usually applied in clinical practice to optimize GM dosing regimens [10]. Several studies have investigated the influence of daily dosing frequency on efficacy and adverse effects in aminoglycoside antimicrobial agents [11–13]. In addition, several clinical studies have attempted to investigate alternative drugs [14] and shorten the administration period [15] to avoid renal dysfunction due to the synergistic effects of GM administration. However, no study has specifically demonstrated a relationship between daily dosing and the occurrence of renal dysfunction in the GM treatment for synergistic effects. Therefore, it is essential to evaluate whether the different dosing regimen of GM at the start of administration affects renal dysfunction in the interval before dose correction by TDM. In the present study, we aimed to investigate the impact of once-daily and multiple-daily GM dosing on the achievement of target trough concentrations and incidence of acute kidney injury (AKI).

## Methods

### Study design and patients

This retrospective observational study was performed at Shizuoka General Hospital, Shizuoka, Japan. The ethics committee of Shizuoka General Hospital approved the study protocol (approval number: SGHIRB#2,021,095). The study participants were included according to the following inclusion criteria: admitted to Shizuoka General Hospital between January 2016 and January 2022, treated with GM in combination with other antimicrobial agents, and with at least one trough concentration. In addition, the exclusion criteria were patients younger than 18 years, patients with an estimated glomerular filtration rate (eGFR) of less than 10 mL/min/1.73 m^2^, patients receiving renal replacement therapy, and patients receiving GM for four days or less [16].

### Outcomes and variables

The outcomes of this study were the achievement rate of an initial GM trough concentration < 1.0 µg/mL and the incidence of AKI to reflect the impact of the dosing regimen at the start of treatment. AKI was defined according to the Kidney Disease Improving Global Outcomes (KDIGO) guidelines (serum creatinine ≥ 0.3 mg/dL within 48 h of GM initiation or serum creatinine ≥ 1.5-fold increase within seven days) [17].

We collected the following observational and laboratory data: age, sex, weight at the time of GM initiation, body mass index (BMI), serum creatinine, eGFR, GM dosage (once daily, twice daily, and three times daily), number of days of GM administration, GM initial trough concentration, minimum trough concentration within 2 weeks of GM initiation, days to initial GM trough concentration measurement, GM peak concentration, infectious diseases (native valve IE, prosthetic valve IE, device-related infection treated according to IE, and other infectious diseases), the bacteria causing the infection (*Streptococci*, *Staphylococci*, *Enterococci*, and other bacteria), and concomitant drugs that may affect renal function (loop diuretics, vancomycin, colistin, liposomal amphotericin B, non-steroidal anti-inflammatory drugs (NSAIDs), cyclosporine, and tacrolimus) as indicated in the package insert for gentamicin in this country. GM trough concentrations were measured within approximately one week after GM initiation using the enzyme immunoassay with a detection limit of 0.2 µg/mL. Trough concentrations were also measured the following week if treatment was continued. Any samples below the detection limits were assumed to be 0.2 µg/mL. The measuring instrument used was an automatic analyzer (JCA-BM8000 series), and the reagent used was Emit 2000 gentamicin assay.

### Statistical analysis

We compared the GM once-daily dosing and multiple-daily dosing groups or patients with and without AKI using the Wilcoxon rank-sum test for continuous variables and the Fisher’s exact test for categorical variables. In addition, logistic regression analysis was conducted to estimate the odds ratios and their corresponding 95% confidence intervals (CIs) for the incidence of AKI, and receiver operating characteristic (ROC) curve analysis was performed to estimate the predictability of GM trough concentrations for AKI. Finally, Pearson’s correlation and linear regression analyses were performed to evaluate a linear association between GM trough concentration and relative change in serum creatinine within seven days of GM administration. Statistical significance was set at *P* < 0.05. All statistical analyses were conducted using R statistical software (version 4.2.1; R Foundation for Statistical Computing, Vienna, Austria).

## Results

### Characteristics of study participants

We included 35 patients in the study. Table 1 shows the characteristics of patients between initial daily dosing of GM. Among the study participants, 22 patients initially received once-daily GM dosing, 13 received multiple-daily GM dosing (one patient received twice-daily GM dosing, and 12 received GM dosing thrice daily). At the start of GM treatment, there were no significant differences in age, sex, weight, BMI, renal function, daily GM dosage, and concomitant medications between patients with once-daily GM dosing and those with multiple-daily GM dosing (Table 1). GM trough concentrations were measured at a median of 4 days after GM administration in the once-daily and multiple-daily dosing groups. The site of infection and causative organisms are shown in Supplemental Digital Content (see Table S1). In addition, 29 of the 35 patients had their peak concentrations measured at the time of the initial blood concentration assessment. The median peak concentration for the once-daily group (*n* = 19) was 12.3 (interquartile range: 9.2, 16.2) µg/mL, while that for the multiple-dose group (*n* = 10) was 4.7 (3.5, 6.3) µg/mL.

**Table 1:** Comparison of demographic and clinical characteristics of study participants between once-daily dosing and multiple daily dosing groups

	Initial daily dosing of gentamicin		*P* value
	Once daily (*n* = 22)	Multiple daily (*n* = 13)	
Sex, Male	18 (82%)	7 (54%)	0.123
Age (years)	72 (60, 77)	67 (56, 75)	0.384
Weight (kg)	51.8 (43.4, 56.9)	54.9 (47.0, 58.1)	0.433
BMI (kg/m^2^)	19.8 (16.4, 22.3)	21.3 (19.6, 22.6)	0.167
Treatment of gentamicin			
Initial daily dosage (mg/kg)	2.80 (2.30, 4.34)	2.96 (2.40, 3.20)	0.966
Duration of administration (days)	11 (7.5, 13)	15 (15, 22)	0.001
Days to initial trough measurement (days)	4 (3, 5)	4 (3, 5)	0.941
Renal function			
Baseline serum creatinine (mg/dL)	0.75 (0.56, 0.88)	0.72 (0.56, 0.99)	0.768
Baseline eGFR (mL/min/1.73m^2^)	73.0 (66.0, 104.5)	80.0 (67.0, 102.0)	0.781
Drugs at risk for renal impairment			
Loop diuretics	9 (41%)	5 (38%)	1.000
NSAIDs	5 (23%)	7 (54%)	0.079

Data are shown as frequency (percentages) or median (interquartile range)

BMI, body mass index; eGFR, estimated glomerular filtration rate; NSAIDs, non-steroidal anti-inflammatory drugs

### Influence of daily dosing frequency on trough concentrations of gentamicin

**Fig. 1:**
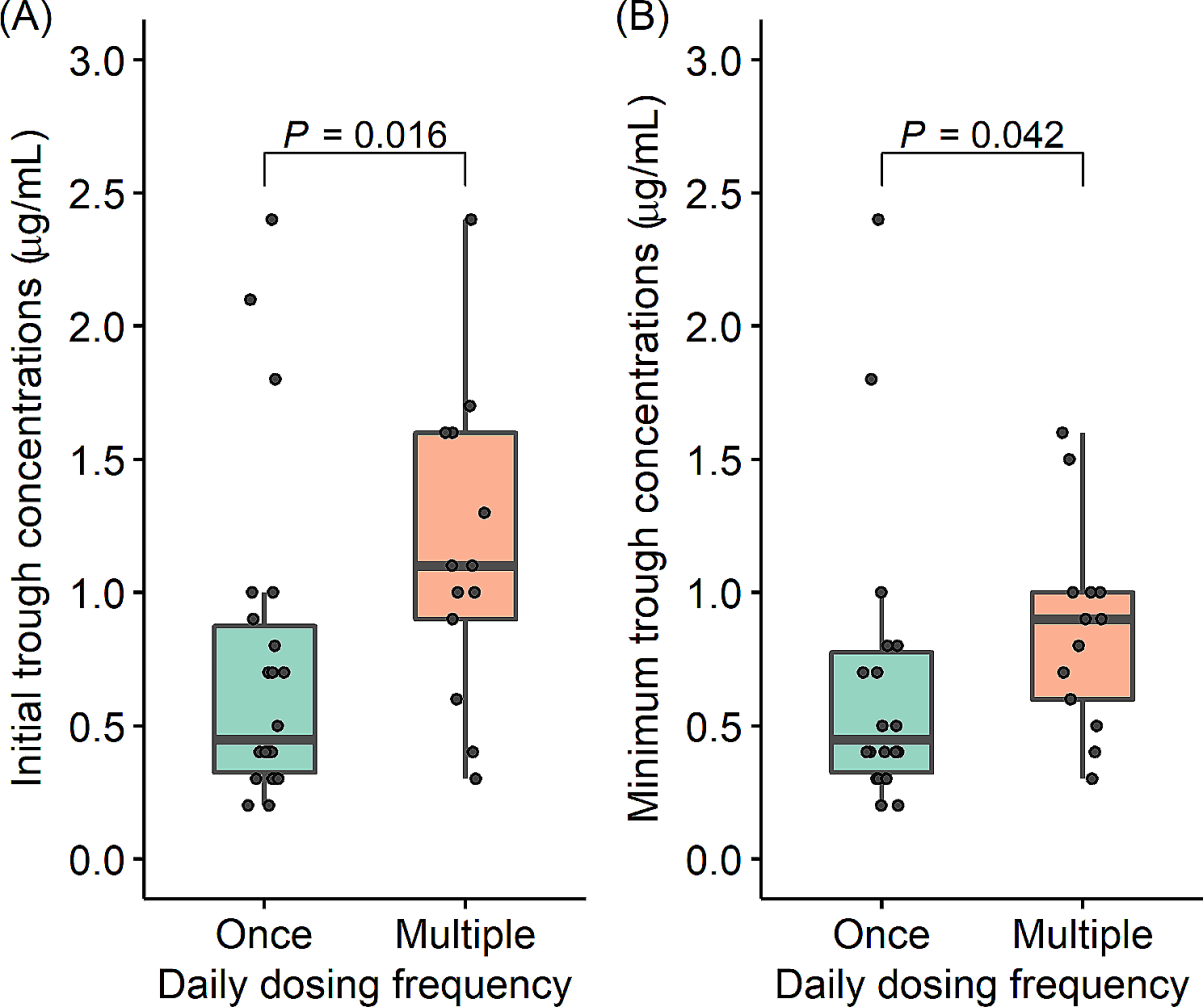
Effect of daily dosing frequency on gentamicin trough concentration. **(A)** Comparison of the initial trough concentrations of serum gentamicin between the once-daily dosing and multiple-daily dosing groups. **(B)** Comparison of the minimum trough concentration

The median of initial GM trough concentrations was 0.45 (interquartile range; 0.33, 0.88) µg/mL and 1.10 (0.90, 1.60) µg/mL in the group of GM once-daily dosing and the group of multiple-daily dosing, respectively. The multiple daily dosing group had significantly higher trough concentrations (*P* = 0.016; Fig. 1A). The frequency of patients with an initial GM trough concentration lower than 1.0 µg/mL was 77% (17/22) in the once-daily group and 31% (4/13) in the multiple-daily group (*P* = 0.012). At our institute, dose optimization is performed using TDM after checking the initial GM trough concentrations. The minimum trough concentration within 2 weeks of GM initiation was 0.45 (0.33, 0.78) µg/mL in the once-daily group and 0.90 (0.60, 1.00) µg/mL in the multiple daily groups, which was still significantly higher in the multiple-daily dosing group *(P* = 0.042, Fig. 1B). However, there were no significant differences in the frequency of minimum trough concentrations lower than 1.0 µg/mL between the once-daily and multiple-daily groups [82% (18/22) vs. 62% (8/13), *P* = 0.243].

### Association with the incidence of acute kidney injury

**Fig. 2:**
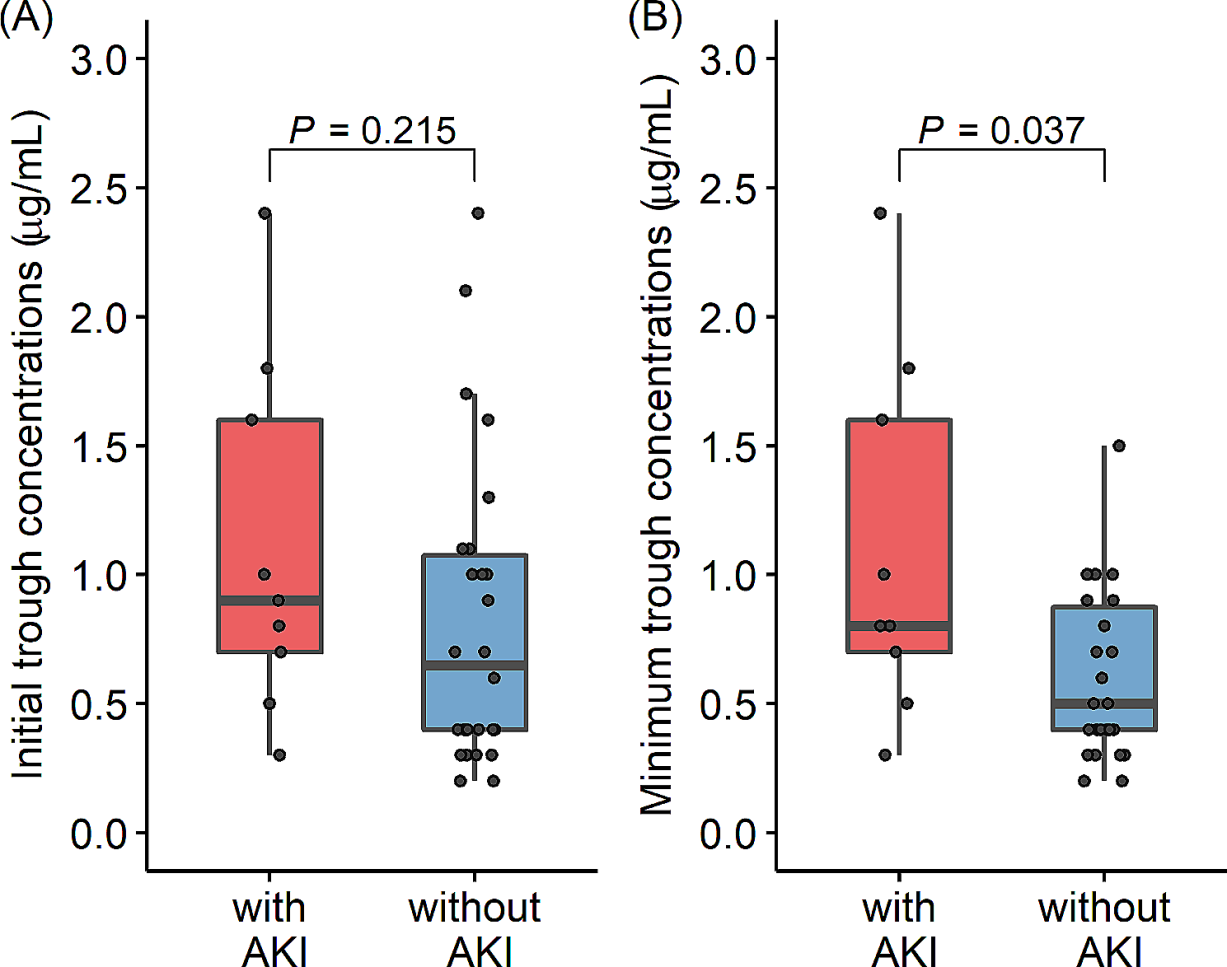
Associations between gentamicin trough concentration and the incidence of acute kidney injury. **(A)** Comparison of the initial serum gentamicin trough concentrations between patients with and without acute kidney injury. **(B)** Comparison of the minimum trough concentration

Nine patients (26%) developed AKI after GM treatment initiation. There were no significant differences in age, sex, weight, BMI, renal function at the start of GM treatment, days to initial GM trough concentration measurement, infectious diseases, or concomitant medications between the patients with and without AKI (Table 2). In addition, we evaluated the association between the daily GM dosing frequency and AKI. However, the incidence rate of AKI was not significantly different between participants with once-daily GM dosing and those with multiple-daily GM dosing (Table 2). The initial GM trough concentrations were slightly higher in patients with AKI than in those without AKI; however, this difference was not statistically significant [median (interquartile range):0.90 (0.70, 1.60) µg/mL vs. 0.65 (0.40, 1.08) µg/mL, *P* = 0.215, Fig. 2A]. In contrast, the minimum trough concentration within 2 weeks of GM initiation was significantly higher in patients with AKI than in patients without AKI [0.80 (0.70, 1.60) µg/mL vs. 0.50 (0.40, 0.88) µg/mL, *P* = 0.037, Fig. 2B]. The site of infection, causative organism, and concomitant antimicrobials are shown in the Supplementary Digital Content (see Table S2).

**Table 2:** Comparison of study participants with and without acute kidney injury (AKI)

	With AKI (*n* = 9)	Without AKI (*n* = 26)	*P* value
Sex, Male	6 (67%)	19 (73%)	0.694
Age (years)	76 (69, 78)	69 (57, 75)	0.190
Weight (kg)	49.6 (49.2, 59.1)	53.4 (46.6, 56.9)	0.610
BMI (kg/m^2^)	19.9 (19.5, 21.5)	20.1 (18.1, 24.1)	0.664
Treatment of gentamicin			
Once daily dosing	8 (89%)	14 (54%)	0.109
Multiple daily dosing	1 (11%)	12 (46%)	-
Initial daily dosage (mg/kg)	2.50 (2.40, 3.00)	2.98 (2.30, 4.29)	0.180
Duration of administration (days)	13 (6, 15)	13 (10, 15)	0.648
Days to initial trough measurement (days)	4 (4, 6)	4 (3, 5)	0.097
Renal function			
Baseline serum creatinine (mg/dL)	0.63 (0.58, 0.94)	0.76 (0.55, 0.90)	0.956
Baseline eGFR (mL/min/1.73m^2^)	78.0 (61.0, 85.0)	77.5 (67.0, 107.3)	0.521
Drugs at risk for renal impairment	7 (78%)	18 (69%)	1.000
Loop diuretics	5 (56%)	9 (35%)	0.432
NSAIDs	2 (22%)	10 (38%)	0.450

Data are shown as frequency (percentages) or median (interquartile range)

AKI, acute kidney injury; BMI, body mass index; eGFR, estimated glomerular filtration rate; NSAIDs, non-steroidal anti-inflammatory drugs

### Association of trough concentrations of gentamicin on the change of renal function

**Fig. 3:**
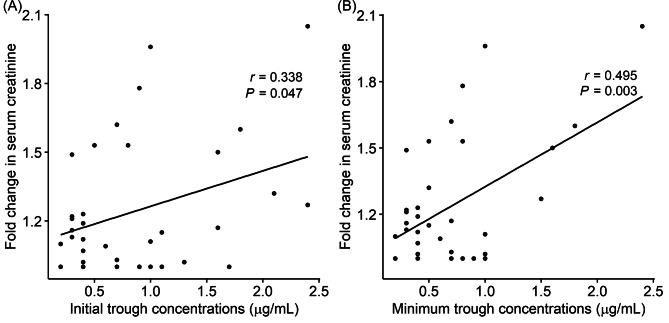
Association of gentamicin trough concentrations with changes in renal function. **(A)** Correlation between the initial trough concentrations of serum gentamicin and fold change in serum creatinine within seven days after gentamicin administration. **(B)** Correlation between the minimum trough concentration and the fold-change in serum creatinine levels

**Fig. 4:**
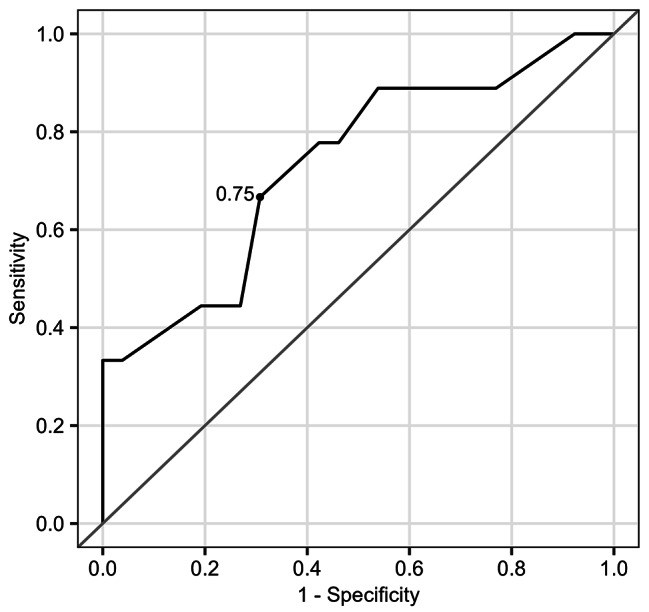
Receiver operating characteristic (ROC) curve for predicting patients with acute kidney injury using minimum trough concentration

Because there was no significant influence of daily dosing frequency on the incidence rate of AKI, we evaluated the association between GM trough concentrations and changes in serum creatinine levels. The relative change in serum creatinine within seven days of GM administration was significantly correlated with the initial GM trough concentrations (*r* = 0.338, *P* = 0.047, Fig. 3A) and minimum GM trough concentration (*r* = 0.495, *P* = 0.003, Fig. 3B). We confirmed these relationships after adjusting for the influence of concomitant drugs that may affect renal function, using multivariate linear regression analysis. The minimum GM trough concentrations [coefficient (*β*) = 0.28 (95% CI; 0.10, 0.47), *P* = 0.004], but not initial trough concentration [*β* = 0.15 (− 0.01, 0.30), *P* = 0.065] maintain statistically significant relationship to the relative change in serum creatinine. Finally, we evaluated the association between minimum GM trough concentration and the incidence of AKI. In logistic regression analysis, the odds ratio for AKI was 9.2 (95% CI; 1.3 to 65.5, *P* = 0.026) per 1.0 µg/mL increase in the minimum GM trough concentrations. The ROC curve analysis revealed that the minimum GM trough concentrations could predict the incidence of AKI with an AUC of 0.73 (95% CI; 0.53, 0.94), a sensitivity of 67%, and a specificity of 69%. The best cut-off value of minimum GM trough concentrations was 0.75 µg/mL to predict the incidence of AKI (Fig. 4).

## Discussion

In the present study, we demonstrated that a higher trough concentration of GM is a risk factor for the incidence of AKI. Although this study did not show a direct influence of daily dosing frequency on the incidence of AKI, patients with multiple-daily dosing had significantly higher trough concentrations than those with once-daily dosing. These results suggested that multiple daily doses of GM may contribute to the development of AKI. In contrast, we demonstrated that the minimum trough concentration of GM, but not the initial trough concentration, was associated with AKI development. These results suggest that it is important to maintain sufficiently low trough concentrations through dose optimization using TDM. Although AKI was used as the primary endpoint in this study to evaluate the association between the initiation dose regimen and renal dysfunction, performing TDM and monitoring serum creatinine levels regularly during GM administration is important.

In implementing TDM, it should be considered that guidelines for IE treatment in each country recommended maintaining a GM trough concentration below 1.0 µg/mL. A high trough GM concentration is associated with renal dysfunction [8, 18, 19]. In the present study, ROC analysis indicated 0.75 µg/mL as the best cut-off value for the minimum trough concentration that predicts the development of AKI. This result seems comparable to the guideline recommendation level and is therefore reasonable. Moreover, we confirmed that there was no difference in the time interval for the first measurement of the GM trough concentration in patients with and without AKI. In both groups, the initial trough concentrations were measured after a median of 4 days. Since the first trough concentration blood sampling after GM administration reaches a steady state approximately 16 h after the first dose [20], it is possible that AKI can be avoided by earlier blood concentration monitoring and implementation of TDM. However, blood levels of aminoglycoside antimicrobial agents are considered outsourced in many facilities [21] and appropriate scheduling is necessary.

Aminoglycoside antimicrobial agents such as GM exhibit therapeutic activity in a concentration-dependent manner [22], and once-daily dosing is currently recommended [23], which is considered the same when synergistic effects are expected [11]. The ESC [4] and JCS guidelines [6] recommend a once-daily method based on clinical studies that showed no significant difference in the efficacy and incidence of renal dysfunction between the once-daily and twice-daily methods of GM administration [12]. However, the AHA guidelines [3] recommend a multiple-dose regimen for treating *Enterococcal* IE, based on in vitro and experimental animal data [24]. The AHA and ESC guidelines provide different recommendations for the peak concentration of GM. The AHA guidelines suggest a peak concentration of 3–4 µg/mL for multiple daily dosing, while the ESC guidelines recommend a peak concentration of 10–12 µg/mL for once daily dosing. However, the AHA does not provide guidance on peak concentration for once-daily dosing, and the ESC does not provide guidance on peak concentration for multiple-daily dosing. The relationship between peak concentration and therapeutic effect is controversial, especially when synergistic effects are considered. Therefore, the basis for the guideline recommendation appears to be inadequate. In the case of bacterial meningitis caused by *Listeria monocytogenes*, a combination of penicillin or ampicillin plus GM is considered, and 5 mg/kg/day in three divided doses is recommended [25]. Although the Bacterial Meningitis Guideline does not list a recommended blood concentration for *Listeria monocytogenes*, we believe that a trough concentration < 1.0 µg/mL is desirable to prevent the development of renal dysfunction, regardless of dosage and method of administration [8]. Based on the results of this study, in which many patients failed to clear the initial trough concentration < 1.0 µg/mL with multiple daily doses of approximately 3 mg/kg/day, it is possible that the target value may not be met even with 5 mg/kg/day divided into three doses. In addition, a cautionary statement has been made regarding the concomitant use of GM owing to the risk of renal dysfunction [26]. As with IE, GM’s efficacy and side effects of GM should be verified in future clinical studies.

Serum GM concentrations are known to vary according to various factors, which may influence the development of AKI. However, in this study, the clinical characteristics of patients, including age, sex, and BMI, were not associated with the incidence of AKI. In contrast, although the duration of GM administration was no different between patients with and without AKI, it was longer in the multiple-daily dosing group than in the once-daily dosing group. One possible reason is that more patients in the multiple-daily dosing group had IE due to *Enterococci*, for which more extended dosing is recommended. Another reason may be that the once-daily group included infections such as *Pseudomonas aeruginosa*, which are not established in the recommended dosing period.

This study has several limitations. The first limitation is the sample size. As this observational study was performed at a single center, the number of participants receiving GM treatment was limited. The fact that there was no statistically significant difference in the incidence of AKI according to the frequency of daily dosing is most likely due to the limited sample size. Second, this study was unable to assess the potential risks, including comorbidities and their severity associated with increased GM trough concentrations and the development of AKI. Finally, this study did not evaluate the efficacy of the antibiotic therapy. Many clinical experts are concerned about the effect of different GM administration methods on efficacy. However, with the sample size of 35 patients in this study, it was difficult to fully assess its influence on efficacy. Further observational studies and clinical trials are required to develop an appropriate GM dosing regimen to reduce the incidence of renal dysfunction.

## Conclusions

A once-daily GM dosing regimen with synergistic effects is a helpful strategy for achieving a lower trough concentration. Since high trough concentrations are strongly implicated in the development of AKI, it may be essential to consider a once-daily dosing regimen or to implement TDM earlier in the treatment period to monitor the GM trough concentration.

### Electronic supplementary material

Below is the link to the electronic supplementary material.

Supplementary Material 1: Table S1. Comparison of site of infection and causative organisms between once-daily dosing and multiple-daily dosing groups. Table S2. Comparison of site of infection and causative organisms between with and without AKI.

## Data Availability

All data generated or analyzed during this study are included in this published article.

## Abbreviations

AHA: American Heart Association
AKI: Acute kidney injury
BSAC: British Society for Antimicrobial Chemotherapy
BMI: Body mass index
CI: Confidence interval
eGFR: Estimated glomerular filtration rate
ESC: European Society of Cardiology
GM: Gentamicin
IE: Infective endocarditis
JCS: Japanese Circulation Society
NSAID: Non-steroidal anti-inflammatory drug
ROC: Receiver operating characteristic
TDM: Therapeutic drug monitoring
