# Supplementary material for: Impact of once-daily versus multiple-daily dosing of gentamicin on the incidence of acute kidney injury in patients treated with synergistic combinations of antibiotics
Source: J Pharm Health Care Sci. 2024 Jul 15;10:40. doi: 10.1186/s40780-024-00360-y (PMC11247752; doi:10.1186/s40780-024-00360-y)
Supplement: Supplementary file 1 — Supplementary Material 1: Table S1. Comparison of site of infection and causative organisms between once-daily dosing and multiple-daily dosing groups. Table S2. Comparison of site of infection and causative organisms between with and without AKI. [file 40780_2024_360_MOESM1_ESM.docx]

**Supplementary material**

Table S1. Comparison of site of infection and causative organisms between once-daily dosing and multiple daily dosing groups

|  | Initial daily dosing of gentamicin | | *P* value |
| --- | --- | --- | --- |
|  | Once daily  (n = 22) | Multiple daily  (n = 13) |  |
| Infectious disease |  |  |  |
| Native valve IE | 7 (32%) | 6 (46%) | 0.480 |
| Prosthetic valve IE | 3 (14%) | 2 (15%) | 1.000 |
| Device infection similar to IE | 1 (5%) | 2 (15%) | 0.541 |
| Infectious aneurysm | 1 (5%) | 0 (0%) | 1.000 |
| Pneumonia | 6 (27%) | 1 (8%) | 0.220 |
| Cholangitis | 2 (9%) | 0 (0%) | 0.519 |
| Bacterial meningitis | 1 (5%) | 1 (8%) | 1.000 |
| Prosthetic joint infection | 0 (0%) | 1 (8%) | 0.371 |
| Osteomyelitis | 1 (5%) | 0 (0%) | 1.000 |
| Inflammatory bacteria |  |  |  |
| Strains penicillin-susceptible (MIC ≤0.125 mg/L) Oral and digestive *Streptococci* | 7 (32%) | 2 (15%) | 0.431 |
| Strains relatively resistant to penicillin (MIC 0.25–2 mg/L) Oral and digestive *Streptococci* | 1 (5%) | 1 (8%) | 1.000 |
| Methicillin-susceptible. *Staphylococcus aureus* | 0 (0%) | 1 (8%) | 0.371 |
| Methicillin-resistant *Staphylococcus aureus* | 1 (5%) | 0 (0%) | 1.000 |
| *Enterococcus faecalis* | 0 (0%) | 3 (23%) | 0.044 |
| *Enterococcus faecium* | 0 (0%) | 1 (8%) | 0.371 |
| *Abiotrophia defectiva* | 0 (0%) | 1 (8%) | 0.371 |
| *Pseudomonas aeruginosa* | 8 (36%) | 1 (8%) | 0.109 |
| *Listeria monocytogenes* | 1 (5%) | 1 (8%) | 1.000 |
| *Corynebacterium striatum* | 0 (0%) | 1 (8%) | 0.371 |
| *Klebsiella aerogenes* | 1 (5%) | 0 (0%) | 1.000 |
| *Helicobacter cinaedi* | 1 (5%) | 0 (0%) | 1.000 |
| *Aerococcus urinae* | 1 (5%) | 0 (0%) | 1.000 |
| *Campylobacter fetus* | 1 (5%) | 0 (0%) | 1.000 |
| Unknown pathogen | 0 (0%) | 1 (8%) | 0.371 |
| Concomitant antibiotics |  |  |  |
| Penicillin G | 5 (23%) | 1 (8%) | 0.377 |
| Ampicillin | 4 (18%) | 7 (54%) | 0.057 |
| Ampicillin/Sulbactam | 2 (9%) | 0 (0%) | 0.519 |
| Piperacillin/Tazobactam | 3 (14%) | 1 (8%) | 1.000 |
| Cefazoline | 1 (5%) | 2 (15%) | 0.541 |
| Ceftriaxone | 4 (18%) | 2 (15%) | 1.000 |
| Ceftazidime | 1 (5%) | 0 (0%) | 1.000 |
| Cefepime | 3 (14%) | 1 (8%) | 1.000 |
| Meropenem | 2 (9%) | 0 (0%) | 0.519 |
| Vancomycin | 6 (27%) | 5 (38%) | 0.708 |
| Clindamycin | 2 (9%) | 0 (0%) | 0.519 |
| Levofloxacin | 2 (9%) | 1 (8%) | 1.000 |
| Minocycline | 0 (0%) | 1 (8%) | 0.371 |
| Clarithromycin | 1 (5%) | 0 (0%) | 1.000 |
| Metronidazole | 1 (5%) | 0 (0%) | 1.000 |
| Rifampicin | 1 (5%) | 0 (0%) | 1.000 |
| Tigecycline | 1 (5%) | 0 (0%) | 1.000 |
| Colistin | 2 (9%) | 0 (0%) | 0.519 |

Data are shown as frequency (percentages) or median (interquartile range).

IE, infective endocarditis.

Table S2. Comparison of study participants with and without acute kidney injury (AKI)

|  | with AKI  (n = 9) | without AKI  (n = 26) | *P* value |
| --- | --- | --- | --- |
| Infectious disease |  |  |  |
| Native valve IE | 3 (33%) | 10 (38%) | 1.000 |
| Prosthetic valve IE | 2 (22%) | 3 (12%) | 0.586 |
| Device infection similar to IE | 1 (11%) | 2 (8%) | 1.000 |
| Infectious aneurysm | 0 (0%) | 1 (4%) | 1.000 |
| Pneumonia | 0 (0%) | 7 (27%) | 0.153 |
| Cholangitis | 1 (11%) | 1 (4%) | 0.454 |
| Bacterial meningitis | 1 (11%) | 1 (4%) | 0.454 |
| Prosthetic joint infection | 0 (0%) | 1 (4%) | 1.000 |
| Osteomyelitis | 1 (11%) | 0 (0%) | 0.257 |
| Inflammatory bacteria |  |  |  |
| Strains penicillin-susceptible (MIC ≤0.125 mg/L) Oral and digestive *Streptococci* | 5 (56%) | 4 (15%) | 0.030 |
| Strains relatively resistant to penicillin (MIC 0.25–2 mg/L) Oral and digestive *Streptococci* | 0 (0%) | 2 (8%) | 1.000 |
| Methicillin-susceptible *Staphylococcus aureus* | 0 (0%) | 1 (4%) | 1.000 |
| Methicillin-resistant *Staphylococcus aureus* | 1 (11%) | 0 (0%) | 0.257 |
| *Enterococcus faecalis* | 0 (0%) | 3 (12%) | 0.553 |
| *Enterococcus faecium* | 0 (0%) | 1 (4%) | 1.000 |
| *Abiotrophia defectiva* | 0 (0%) | 1 (4%) | 1.000 |
| *Pseudomonas aeruginosa* | 1 (11%) | 8 (31%) | 0.391 |
| *Listeria monocytogenes* | 1 (11%) | 1 (4%) | 0.454 |
| *Corynebacterium striatum* | 0 (0%) | 1 (4%) | 1.000 |
| *Klebsiella aerogenes* | 1 (11%) | 0 (0%) | 0.257 |
| *Helicobacter cinaedi* | 0 (0%) | 1 (4%) | 1.000 |
| *Aerococcus urinae* | 0 (0%) | 1 (4%) | 1.000 |
| *Campylobacter fetus* | 0 (0%) | 1 (4%) | 1.000 |
| Unknown pathogen | 0 (0%) | 1 (4%) | 1.000 |
| Concomitant antibiotics |  |  |  |
| Penicillin G | 2 (22%) | 4 (15%) | 0.635 |
| Ampicillin | 4 (44%) | 7 (27%) | 0.416 |
| Ampicillin/Sulbactam | 1 (11%) | 1 (4%) | 0.454 |
| Piperacillin/Tazobactam | 2 (22%) | 2 (8%) | 0.268 |
| Cefazoline | 1 (11%) | 2 (8%) | 1.000 |
| Ceftriaxone | 1 (11%) | 5 (19%) | 1.000 |
| Ceftazidime | 0 (0%) | 1 (4%) | 1.000 |
| Cefepime | 0 (0%) | 4 (15%) | 0.553 |
| Meropenem | 0 (0%) | 2 (8%) | 1.000 |
| Vancomycin | 3 (33%) | 8 (31%) | 1.000 |
| Clindamycin | 2 (22%) | 0 (0%) | 0.061 |
| Levofloxacin | 1 (11%) | 2 (8%) | 1.000 |
| Minocycline | 0 (0%) | 1 (4%) | 1.000 |
| Clarithromycin | 0 (0%) | 1 (4%) | 1.000 |
| Metronidazole | 0 (0%) | 1 (4%) | 1.000 |
| Rifampicin | 1 (11%) | 0 (0%) | 0.257 |
| Tigecycline | 1 (11%) | 0 (0%) | 0.257 |
| Colistin | 2 (22%) | 0 (0%) | 0.061 |

Data are shown as frequency (percentages) or median (interquartile range).

AKI, acute kidney injury; IE, infective endocarditis.
